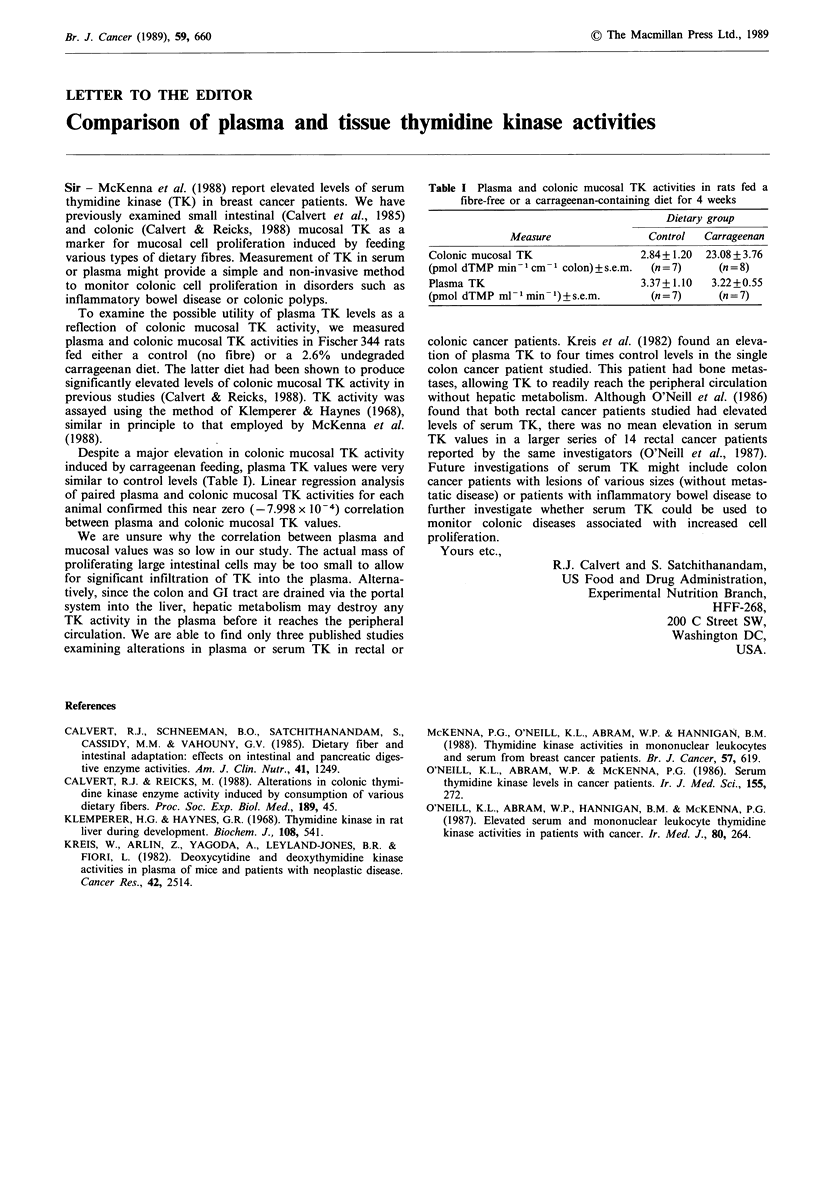# Comparison of plasma and tissue thymidine kinase activities.

**DOI:** 10.1038/bjc.1989.135

**Published:** 1989-04

**Authors:** R. J. Calvert, S. Satchithanandam


 © The Macmillan Press Ltd., 1989

### LETTER TO THE EDITOR

### Comparison of plasma and tissue thymidine kinase activities

**Sir** – McKenna *et al.* (1988) report elevated levels of serum thymidine kinase (TK) in breast cancer patients. We have previously examined small intestinal (Calvert *et al.*, 1985) and colonic (Calvert & Reicks, 1988) mucosal TK as a marker for mucosal cell proliferation induced by feeding various types of dietary fibres. Measurement of TK in serum or plasma might provide a simple and non-invasive method to monitor colonic cell proliferation in disorders such as inflammatory bowel disease or colonic polyps.

To examine the possible utility of plasma TK levels as a reflection of colonic mucosal TK activity, we measured plasma and colonic mucosal TK activities in Fischer 344 rats fed either a control (no fibre) or a 2.6% undegraded carrageenan diet. The latter diet had been shown to produce significantly elevated levels of colonic mucosal TK activity in previous studies (Calvert & Reicks, 1988). TK activity was assayed using the method of Klemperer & Haynes (1968), similar in principle to that employed by McKenna *et al.* (1988).

Despite a major elevation in colonic mucosal TK activity induced by carrageenan feeding, plasma TK values were very similar to control levels (Table I). Linear regression analysis of paired plasma and colonic mucosal TK activities for each animal confirmed this near zero ($-7.998 \times 10^{-4}$) correlation between plasma and colonic mucosal TK values.

We are unsure why the correlation between plasma and mucosal values was so low in our study. The actual mass of proliferating large intestinal cells may be too small to allow for significant infiltration of TK into the plasma. Alternatively, since the colon and GI tract are drained via the portal system into the liver, hepatic metabolism may destroy any TK activity in the plasma before it reaches the peripheral circulation. We are able to find only three published studies examining alterations in plasma or serum TK in rectal or colonic cancer patients. Kreis *et al.* (1982) found an elevation of plasma TK to four times control levels in the single colon cancer patient studied. This patient had bone metastases, allowing TK to readily reach the peripheral circulation without hepatic metabolism. Although O'Neill *et al.* (1986) found that both rectal cancer patients studied had elevated levels of serum TK, there was no mean elevation in serum TK values in a larger series of 14 rectal cancer patients reported by the same investigators (O'Neill *et al.*, 1987). Future investigations of serum TK might include colon cancer patients with lesions of various sizes (without metastatic disease) or patients with inflammatory bowel disease to further investigate whether serum TK could be used to monitor colonic diseases associated with increased cell proliferation.

**Table I** Plasma and colonic mucosal TK activities in rats fed a fibre-free or a carrageenan-containing diet for 4 weeks

| | *Dietary group* | |
|---|---|---|
| *Measure* | *Control* | *Carrageenan* |
| Colonic mucosal TK (pmol dTMP $min^{-1} cm^{-1}$ colon) ± s.e.m. | 2.84 ± 1.20 ($n$=7) | 23.08 ± 3.76 ($n$=8) |
| Plasma TK (pmol dTMP $ml^{-1} min^{-1}$) ± s.e.m. | 3.37 ± 1.10 ($n$=7) | 3.22 ± 0.55 ($n$=7) |

Yours etc.,

R.J. Calvert and S. Satchithanandam,
US Food and Drug Administration,
Experimental Nutrition Branch,
HFF-268,
200 C Street SW,
Washington DC,
USA.

### References

CALVERT, R.J., SCHNEEMAN, B.O., SATCHITHANANDAM, S., CASSIDY, M.M. & VAHOUNY, G.V. (1985). Dietary fiber and intestinal adaptation: effects on intestinal and pancreatic digestive enzyme activities. *Am. J. Clin. Nutr.*, **41**, 1249.

CALVERT, R.J. & REICKS, M. (1988). Alterations in colonic thymidine kinase enzyme activity induced by consumption of various dietary fibers. *Proc. Soc. Exp. Biol. Med.*, **189**, 45.

KLEMPERER, H.G. & HAYNES, G.R. (1968). Thymidine kinase in rat liver during development. *Biochem. J.*, **108**, 541.

KREIS, W., ARLIN, Z., YAGODA, A., LEYLAND-JONES, B.R. & FIORI, L. (1982). Deoxycytidine and deoxythymidine kinase activities in plasma of mice and patients with neoplastic disease. *Cancer Res.*, **42**, 2514.

McKENNA, P.G., O'NEILL, K.L., ABRAM, W.P. & HANNIGAN, B.M. (1988). Thymidine kinase activities in mononuclear leukocytes and serum from breast cancer patients. *Br. J. Cancer*, **57**, 619.

O'NEILL, K.L., ABRAM, W.P. & McKENNA, P.G. (1986). Serum thymidine kinase levels in cancer patients. *Ir. J. Med. Sci.*, **155**, 272.

O'NEILL, K.L., ABRAM, W.P., HANNIGAN, B.M. & McKENNA, P.G. (1987). Elevated serum and mononuclear leukocyte thymidine kinase activities in patients with cancer. *Ir. Med. J.*, **80**, 264.